# Plasmonic Hot-Electron Transfer in Gold-Nanostar-Conjugated Poly(heptazine imide) Photocatalyst

**DOI:** 10.1021/acs.nanolett.6c01897

**Published:** 2026-06-24

**Authors:** Anton Yu. Bykov, Koen Evers, Diptiranjan Paital, Pankaj Sharma, Fang Xie, Anatoly V. Zayats

**Affiliations:** † Nanophotonics Centre, Cavendish Laboratory, Department of Physics, 2152 University of Cambridge, Cambridge CB3 0US, U.K.; ‡ Department of Physics and London Centre for Nanotechnology, 4616 King’s College London, London WC2R 2LS, U.K.; § Department of Materials, 4615 Imperial College London, London SW7 2AZ, U.K.

**Keywords:** plasmonics, hot-electron dynamics, photocatalysis, polymeric carbon nitride

## Abstract

Plasmonic metal–semiconductor hybrid systems have emerged as promising platforms for enhancing photocatalytic efficiency through hot-carrier generation and transfer, with applications in solar energy conversion and chemical synthesis. Here, we investigate ultrafast charge carrier dynamics in a polymeric carbon nitride photocatalyst hybridized with gold nanostars. Using pump–probe spectroscopy, efficient electron scavenging and strong interfacial electronic coupling between gold and a poly­(heptazine imide) matrix was observed, manifesting in significant modifications in the hot-carrier relaxation dynamics. Hot-electron injection from gold to poly­(heptazine imide) with an efficiency of approximately 40% (standard error 12%) was observed, one of the highest reported for metal–semiconductor systems. This enhanced efficiency is attributed to the nanostar morphology, whose sharp features promote momentum relaxation and facilitate interfacial charge transfer. The findings provide direct insight into excitation-dependent electron dynamic processes and highlight the strong potential of carbon nitride-based composites for plasmonically enhanced photocatalysis, offering guidance for the design and optimization of advanced photocatalytic systems.

## Graphical abstract

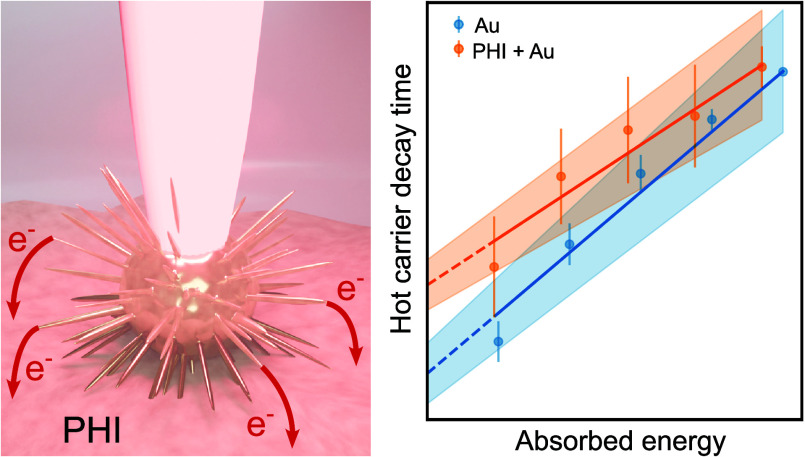

Electron transfer from plasmonic nanoparticles to photocatalysts can significantly enhance the efficiency of solar-driven chemical reactions.^1–3^ Plasmonic nanoparticles support localized surface plasmons (LSPs) that concentrate electromagnetic energy at the nanoscale and generate nonequilibrium (“hot”) charge carriers. These energetic electrons (and/or holes) can be transferred to nearby molecules or semiconductor supports, directly driving interfacial redox chemistry or promoting charge separation and longer-lived carriers. To exploit these effects, various hetero-nanoparticles and metastructures combining plasmonic and catalytic components have been designed and studied.^4^

Among numerous photocatalysts, polymeric carbon nitrides, particularly poly­(heptazine imide) (PHI), have emerged as promising materials for clean energy conversion, carbon dioxide reduction, and as selective organic photoredox catalysis.^5,6^ PHI absorbs blue light (absorption edge 440–460 nm, band gap 2.7–2.8 eV) and generates long-lived charge carriers capable of driving redox reactions.^7^ Recent efforts have been focused on engineering PHI-based heterojunctions, where built-in electric fields promote charge separation and broaden spectral response.^8^ The stability of PHI, the tunable band structure, and compatibility with single-atom catalysts further broaden its applicability in photocatalysis, photoelectrocatalysis, and organic synthesis.^9,10^ PHI adopts a two-dimensional layered ionic structure of imide-bridged heptazine units, with metal cations intercalated in the triangular pore channels; the nature of these cations directly governs the degree of π-conjugation and intrinsic charge carrier mobility in the framework.^11–13^ Edge-terminating cyanamide groups serve as preferential anchoring sites for noble metal cocatalysts, lowering the barrier for interfacial electron transfer and thus overcoming the intrinsic limitations of carrier transport to improve photocatalytic performance.^9,15^

To extend light harvesting across the visible spectrum, PHI can be coupled to plasmonic nanoparticles whose optical absorption and the associated electromagnetic field enhancement are readily tuned through particle size, morphology, and choice of material. In such composites, electron injection from the photoexcited nanoparticles into PHI is a key step that converts broadband plasmonic absorption into usable charge carriers capable of initiating and driving interfacial chemical transformations. In our recent work, we introduced gold nanostars (AuNSs) in a PHI matrix, achieving a plasmonic–polymeric carbon nitride photocatalyst for significantly improved solar-driven peroxide (H_2_O_2_) production.^16^ In this reaction, the reduction of dissolved oxygen by the hot electrons is coupled to harvesting of hot holes via oxidation of isopropanol, which was present in solution as a sacrificial electron donor.

Multiple sharp tips of the nanostars provide strong electromagnetic field enhancement due to both multiple LSPs that cover a broad spectral range from visible to near-IR and the lightning rod effect. These enhanced fields result in hot-electron excitation and are expected to initiate the related electron transfer processes at the Au–PHI interface.^17,18^ In AuNSs-PHI composites, photoluminescence quenching and reduced interfacial charge-transfer resistance indicate more efficient light harvesting, charge separation, and transport than those in a PHI medium alone. A time-dependent density functional theory study of Au clusters adsorbed to PHI further suggests the presence of interfacial excitations in which charge density is displaced across the junction between Au and PHI, consistent with the transfer of the electron density to PHI.^16^ These studies, however, do not directly resolve the ultrafast pathways and competing relaxation and trapping processes that govern hot-carrier injection and charge retention under different excitation conditions (e.g., plasmonic versus PHI interband excitation). Understanding electron transfer in Au-PHI heterostructures is important for the design and optimization of the photocatalytic properties and hence developing new applications.

In this paper, we use femtosecond pump–probe spectroscopy to elucidate the electron excitation and transfer processes in AuNSs-PHI composites and directly demonstrate the presence of electron transport in this system. We observe the apparent quenching of charge trapping in PHI, when excited directly by interband optical excitation of the semiconductor in the presence of gold, indicative of the electron scavenging behavior of AuNSs, and the modification of the hot-carrier dynamics in gold under near-IR resonant plasmonic excitation, which we ascribe to the ultrafast hot-carrier injection in PHI. The latter is likely responsible for enhanced catalytic performance. We quantified the efficiency of the injection process on the order of 40%, one of the largest values reported to date for metal–semiconductor catalytic systems.^19,20^ The results elucidate the hot-carrier behavior in metal–semiconductor hybrid systems under different excitation conditions, which is important for the design and optimization of plasmonically assisted photocatalysts for different operating conditions.

Poly­(heptazine imide) was synthesized by ionothermal polymerization under inert conditions (Figure 1a,b). Gold nanostars (Figure 1d) and AuNSs-PHI composites (Figure 1c) were fabricated using a wet-chemistry approach (see details in the Methods section). Transmission electron microscopy (TEM) images reveal an even distribution of the nanostars on the platelet (Figure 1c). All three studied samples (PHI, AuNSs, and AuNSs-PHI) were deposited on a silica slide to probe their optical properties.

**Figure 1:**
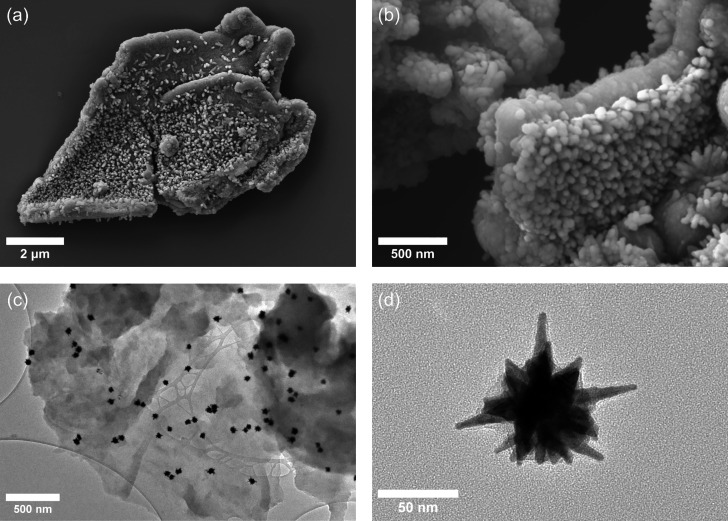
Morphological characterization. (a) Scanning electron microscopy (SEM) image of a PHI flake without the nanostars and (b) its cross-section image. (c, d) TEM images of (c) AuNSs-PHI and (d) a typical Au nanostar.

The optical absorption spectrum of AuNSs on a substrate (Figure 2b) exhibits a broad band centered at approximately 685 nm, which is characteristic of localized surface plasmons arising from both dipolar and multipolar modes associated with the branched morphology of AuNSs.^21^ Pristine PHI flakes exhibit strong ultraviolet absorption below 450 nm with a shoulder peak around 500 nm (Figure 2b), corresponding to its intrinsic π–π* and n−π* electronic transitions, respectively.^22^ Upon integration of AuNSs with PHI, a blue shift of the LSP band from 685 nm to approximately 640 nm is observed (Figure 2b). Although the refractive indices of glass and PHI are close (1.53 and 1.55–1.6, respectively), the shift is interpreted as a result of the increase in the spacing of Au nanostars deposited on PHI compared to the densely packed arrangement on glass, where the coupling between adjacent plasmonic particles redshifts and dampens the resonance. The spectral shift may also be indicative of the interfacial interaction between PHI and the Au nanostars. Photocatalytic experiments indicate the important role of the AuNS interaction with PHI under solar illumination for the enhanced H_2_O_2_ production:^16^ the rate is increased almost 100% with AuNSs-PHI composites compared to bare PHI (Figure 2c).

**Figure 2:**
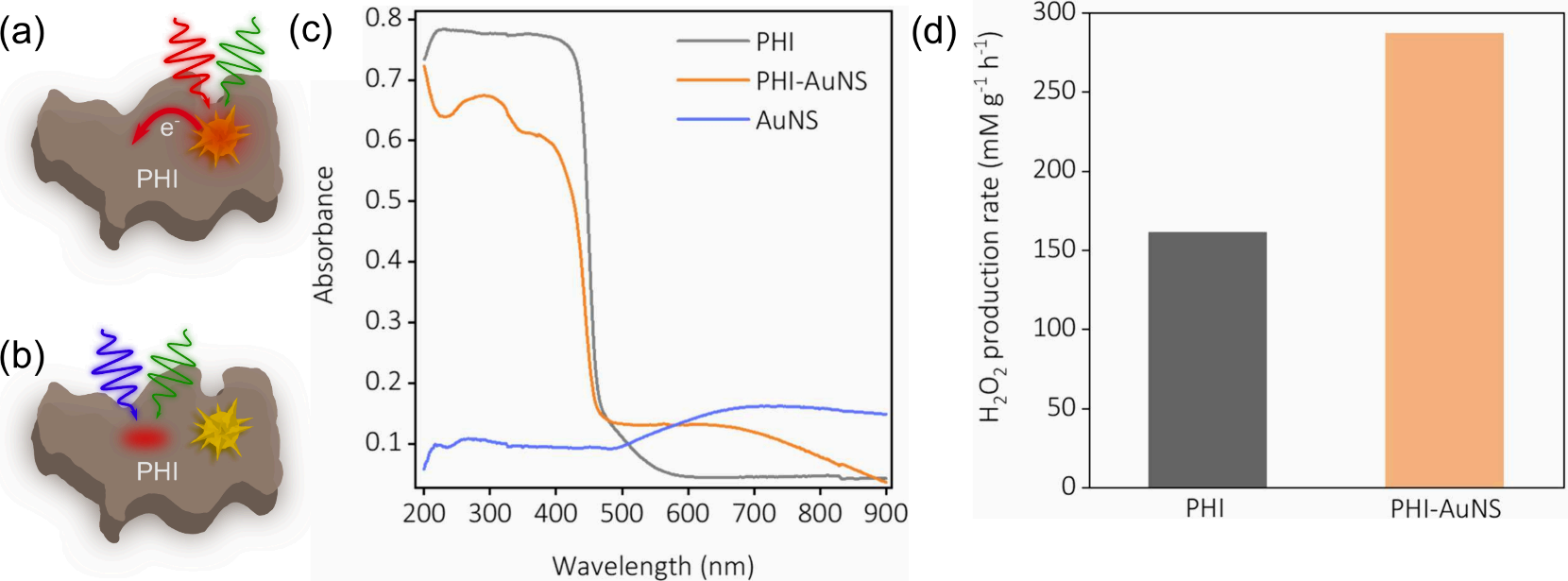
Optical properties. (a, b) Schematics of the processes and experimental geometries: (a) plasmonic NIR excitation and hot electron transfer and (b) direct UV-photoexcitation of PHI; green waveform represents the probe light. (c) Absorption spectra of PHI (black), Au nanostars on a glass substrate (blue), and AuNSs-PHI (orange) samples. (d) Photocatalytic response (H_2_O_2_ production in liquid phase^16^) of bare PHI and AuNSs-PHI composite under simulated solar light.

To understand the hot-electron excitation and transfer in AuNSs-PHI composites, we performed pump–probe spectroscopy (see Methods for details). The approach was highly successful for understanding hot-electron dynamics in plasmonic-metal/cocatalyst-metal photocatalytic systems.^23,24^ In our case of the hybrid plasmonic–semiconductor system, the comparison of the hot-electron dynamics in the case of plasmonic excitation through LSPs and interband excitation of the semiconductor will be instructive. These processes can be clearly separated in the AuNSs-PHI system because of their spectral separation.

An excitation wavelength of 800 nm was chosen to excite plasmonic resonances of Au nanostars, which is close to the maximum of LSP absorption of AuNSs-PHI composites and avoids direct interband absorption in PHI (Figure 2b). The generated hot electrons can then be transferred from the sp band in Au to the PHI. It is important to note that although at this excitation wavelength there is virtually no direct interband absorption in PHI, the photocatalytic experiments under solar illumination (Figure 2c) suggest the existence of a hot-carrier mechanism driving the chemical transformations.^16^

To probe the dynamics of the hot-carrier population in gold, the composite was probed with light at the 514 nm wavelength, which is highly sensitive to both equilibrium and nonequilibrium dynamics in gold,^25,26^ due to the resonant interband transitions around the L high symmetry point in the band structure of gold. Thanks to the inherent dependence of a free-electron heat capacity on temperature, we performed comparative studies of the transient absorption spectra at different excitation fluences (Figure 3(a,b)), from which the hot-electron relaxation times can be extracted. The photoinduced bleach (increased transmission) is observed in the AuNSs on a glass substrate, and the photoinduced absorption (reduced transmission) is observed in the AuNSs-PHI composite. The near-linear dependence of the amplitude of the photoinduced bleach and absorption signals on the excitation fluence can be observed.

**Figure 3:**
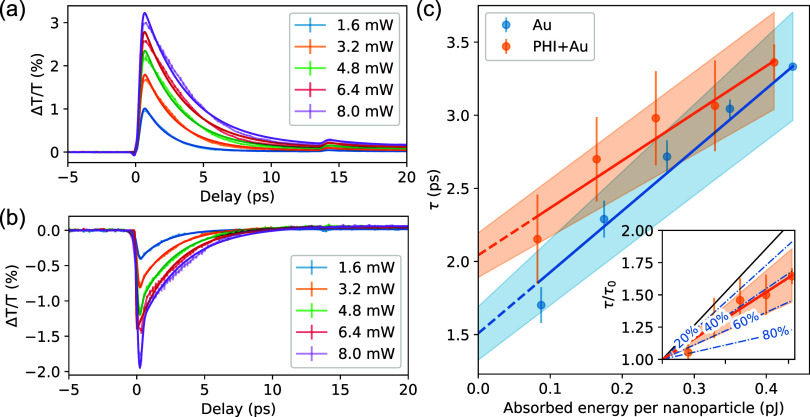
Hot-electron dynamics. (a, b) Transient transmission at the 514 nm wavelength under the 800 nm excitation for different excitation powers measured for (a) gold nanostars and (b) a AuNSs-PHI composite. (c) Power dependence of hot-carrier decay time in AuNSs and the AuNSs-PHI composite: (symbols) measured data, (solid lines) fit, (dashed lines) extrapolation of the fit to low excitation power. The shaded areas represent the 90% confidence interval, the inset shows the theoretical slopes of the power dependence of decay time ratio for the AuNSs-PHI vs AuNSs systems, assuming different efficiencies of hot-carrier injection.

The dependence of the measured decay times on the excitation fluence allows estimating the effective electron–phonon coupling in a gold nanostructures, its dependence on environment, and the degree of carrier injection into an adjacent semiconductor material.^26,27^ In our case, the electrons from the sp band of Au are expected to participate in the interfacial transport so that the appearance of this additional decay channel effectively lowers the apparent excitation fluence, as mostly high-energy nonthermal carriers can travel over the Schottky barrier at ultrafast time scales (not directly captured in the experiment with 100 fs excitation pulses), leaving the colder hot electrons behind. This hot-carrier population in turn decays faster than expected in the absence of injection.^26^ Within the scope of the two-temperature model of hot-carrier dynamics in metal,^3^ the resulting decay times can be estimated as^26,27^

1 $$\tau_{e-ph}=\frac{\gamma T_{l}}{G_{\mathrm{Au}/\mathrm{PHI}}}+\frac{\left(1-P_{i}\right)U_{abs}}{2G_{\mathrm{Au}/\mathrm{PHI}}T_{l}}$$

where *T*_*l*_ is the equilibrium lattice temperature, taken as ambient temperature, γ is the linear proportionality coefficient in the temperature dependence of the free-carrier heat capacity of gold, *G*_*Au*/*PHI*_ is the phenomenological electron–phonon coupling constant, which describes the degree of energy exchange between the electrons and a pool of phonon modes of gold and PHI that the hot-electron ensemble may scatter on, *U*_*abs*_ is the absorbed energy per unit volume of gold, which can be calculated from the measured absorption spectra and the coverage density of AuNSs (see Methods for evaluation of density of AuNSs), and *P*_*i*_ is the electron injection probability from Au to PHI. The higher *P*_*i*_ therefore results in lower maximum electron temperatures and overall faster relaxation. The charge separation produced by the injection relaxes before the next excitation pulse, by recombination through the Schottky barrier.

Linear extrapolation of the power dependence of the hot-carrier decay time data to zero fluence (Figure 3c) reveals lower values of the effective electron–phonon coupling constant in gold nanostars interfaced with PHI, compared to the glass substrate. This behavior indicates a lower phonon heat conductivity of the PHI than a silica substrate, with similar substrate heat conductivity dependences reported in multiple previous hot-carrier studies in metals on substrates and in solutions.^26,28–30^ This effect stems from the reduced role of scattering of hot carriers on optical phonons in the substrate and is used to refine the estimates of injection efficiency. There is a lack of literature data on phonon heat conductivities of disordered PHI networks, and while graphitic carbon nitrides may demonstrate heat conductivities higher than glass,^31^ more similar to PHI polymeric carbon nitrides have values below 1 W mK^–1^,^32^ closer to the values expected from disordered polymers. The obtained values of electron–phonon coupling for Au on glass and PHI are estimated here as *G*_*Au*_ = 1.33 × 10^16^ W m^–3^ K^–1^ and *G*_*Au*/*PHI*_ = 0.97 × 10^16^ W m^–3^ K^–1^, respectively. The electron–phonon constant for gold nanostars on glass is smaller than previously reported bulk values inferred from the measurements of single-crystal gold flakes.^26^ Similar deviations were observed in previous studies of small metal particles and attributed to surface effects, which might be of importance for star-shaped nanoparticles.^33^

Using the determined electron–phonon relaxation rate, one can evaluate *P*_*i*_ from the slope of the power dependence, assuming that no injection is occurring on an insulating substrate. We obtain a significant injection of 40% (90% confidence interval 21–60%), which corroborates the results of photocatalytic studies under visible light illumination. It is important to note that this number represents a fraction of hot-electron energy transferred between AuNSs and PHI, rather than the absolute number of hot electrons transferred.

To further corroborate the ultrafast electronic coupling between PHI and gold nanostars, we investigated the dynamics of hot carriers directly excited in PHI under the excitation at a wavelength of 400 nm, above the band gap of PHI (Figure 4). Although in this regime the hot carriers in gold are also excited, the signals can be separated by considering a time interval of 10s–100s ps, where no distinct ultrafast electronic processes remain in pure gold. In PHI, on the other hand, the characteristic decay in a 100 ps range is expected and has been reported previously, associated with the dynamics of photoexcited electrons and charge trapping.^34^ The subsequent long-lived trap states that survive for up to tens of microseconds were routinely observed with ns-to-μs transient absorption spectroscopy and linked to the superior catalytic performance of carbon nitrides.^35,36^

**Figure 4:**
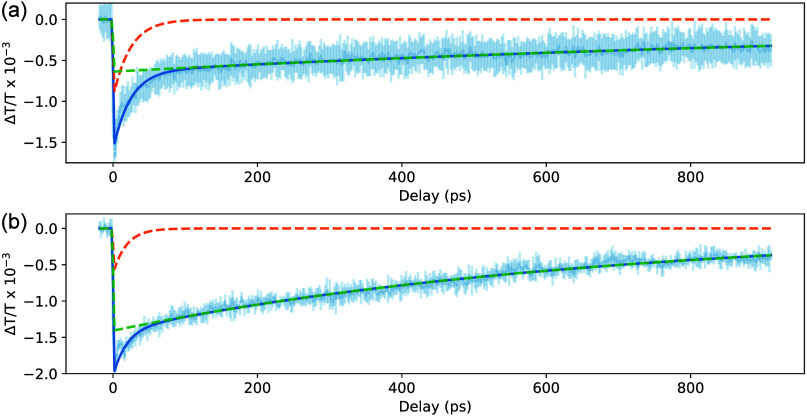
Carrier dynamics in PHI. Transient transmission at the 514 nm wavelength under the 400 nm UV photoexcitation measured in (a) AuNSs-PHI composite and (b) PHI on the glass substrate. Orange and green dashed fits represent short and long decay components, respectively.

Photoinduced absorption is observed under this excitation in a bare PHI which decays on a 100s ps–ns time scale. Similarly to previous studies,^34^ we attribute this transient absorption to the dynamics of photogenerated electron–hole pairs in PHI. While we do not resolve the entire dynamic range of the decay process, the characteristic time scales of this process can be recovered by considering a biexponential decay:^3^

$$\frac{\Delta T}{T}\propto \left[Ae^{-t/\tau_{s}}+Be^{-t/\tau_{l}}\right]\theta (t-t_{0}){\;}^{*}G\left(t\right)$$ 2

where *G*(*t*) is the pump–probe pulse cross-correlation, θ is the Heaviside step function, and * represents the convolution operation. The two characteristic time scales recovered from the measured dependences for pure PHI are 18.5 ± 1.9 ps and 0.68 ± 0.01 ns (Figure 4b). While the longer time scale can be naturally attributed to the charge-trapping process,^34^ we would like to note that the short decay of ≈20 ps has not been reported in transient absorption measurements of carbon nitrides to the best of our knowledge. However, similar time scales have been reported in the time-resolved photoluminescence.^37^

When PHI is interfaced with AuNSs (Figure 4a), the short decay channel remains mostly unaffected (τ_*s*_ = 23.5 ± 1.3 ps), supporting the conclusion that despite the presence of gold, which is also sensitive to excitation at 400 nm, the main contribution to the observed decay is still coming from the photoexcitation of PHI. At the same time, the longer time scale decay channel, observed in pure PHI, quenches and is effectively not seen in the dynamic response of the AuNSs-PHI composite. This observation indicates that the presence of AuNSs provides an efficient sink for the photogenerated electrons in PHI, suppressing the formation of long-lived trapped states, consistent with the previous observations involving the addition of Ag^+^ ions to PHI or PHI hybridization with Pt.^34^ (It should be noted that although the fits are able to recover another very long decay time of 1.34 ± 0.04 ns, the low amplitude and limited temporal span of the measurements make accurate estimates challenging.)

In summary, we have studied carrier dynamics in a AuNSs-PHI nanocomposite, in order to assess the role of hot-electron injection from plasmonic metal as a driver for superior catalytic performance of the combined material system. In pump–probe experiments, the overall electronic coupling between AuNSs and PHI was demonstrated by monitoring picosecond transient absorption of photogenerated hot carriers in PHI, for which gold acts as an electron scavenger. Under near-IR illumination resonant with plasmonic excitations in the system, we observe a clear modification of temporal dynamics of hot electrons in gold that is indicative of hot-carrier injection with an efficiency on the order of 40%. This is one of the highest metal–semiconductor injection efficiencies reported to date and is potentially facilitated by momentum relaxation imposed by the nanostar morphology featuring sharp tips. Despite general difficulties of quantitatively determining the hot-carrier injection efficiencies in this complex and disordered media, the obtained results showcase the high potential of carbon nitrides in hybrid plasmonic–semiconductor photocatalysis and provide guidance for the design and optimization of plasmonic-enabled photocatalysts.

## Methods

### PHI Synthesis

PHI-structured carbon nitride was synthesized using ionothermal polymerization under strict inert conditions following the previously reported method.^16^ A constant Ar flow minimized air and moisture, while a 5:3 urea/KCl–LiCl eutectic mixture was polymerized in a tube furnace. The mixture was first heated at 155 °C for 0.5 h under Ar to remove residual O_2_ and H_2_O, then ramped at 3 °C min^–1^ to 550 °C and maintained for 6 h. The resulting greenish-yellow product was washed with Milli-Q water, filtered, vacuum-dried, ground, and stored in amber vials. This yielded a highly ordered, efficient PHI photocatalyst powder.

### Gold Nanostar Synthesis and Loading on PHI

AuNSs were synthesized as described in ref 16 by introducing 0.4 mL of a gold seed solution of citrate-stabilized gold nanoparticles into a mixture containing 40 μL of 1.0 M hydrochloric acid solution (Titripur, Sigma-Aldrich, 1.09057), 0.18 mL of 25 mM gold­(III) chloride trihydrate (>99.9%, Sigma-Aldrich, 520918), and 20 mL of Milli-Q water. Following this addition, 0.6 mL of 2 mM silver nitrate (>99%, Sigma-Aldrich, 209139) and 0.4 mL of l-ascorbic acid (>99%, Sigma-Aldrich, 255564) were swiftly (within one second of each other) introduced. The outcome of this process was the formation of gold nanostars. Following the synthesis, only trace amounts of metallic Ag are left in the structure, as evidenced from the EXAFS phase analysis, HRTEM *d*-spacing, and XPS described in ref 16. To incorporate AuNSs into the PHI matrix to form a composite, a suspension of 100 mg of PHI in 5 mL of water, which had been stirred for 1 h, was mixed with freshly synthesized AuNSs equal to 0.3 mg gold weight, yielding a 0.3 wt % loading. Subsequently, the mixture was shaken, sonicated, and transferred to a plastic Falcon tube and then rapidly frozen by immersing the tube directly in liquid nitrogen. The removal of water from the frozen sample was achieved over 72 h using a benchtop freeze-drying system (FreeZone 4.5, Labconco).

### Gold Nanostar Density Determination

To estimate the density of AuNSs, we first determined the surface area of each AuNS as 4015 nm^2^ from a TEM image using ImageJ thresholding and reduced it to a circle with a radius of *r* = 35.74 nm. We then calculated the sphere volume $V=\frac{4}{3}\pi r^{3}$ . Using the gold density of 19.32 g cm^–3^, we estimated the weight of a single nanostar to be approximately 3.7 fg. Using the density of PHI (≈1.5 g cm^–3^), the mass of 1 μm^3^ PHI is 1500 fg. At 0.3 wt % gold nanostar loading, this volume contains ≈4.5 fg of gold, corresponding to ≈1.21 nanostars per μm^3^ (or 1.21 × 10^12^ particles per cm^3^). This approximation does not account for structural factors such as porosity and flake roughness, which (through effectively reducing the mass of PHI in a given volume) would slightly decrease the effective nanostar concentration.

### Optical Absorption Measurements

The optical absorption spectra of PHI, AuNSs, and the AuNSs-PHI composite were measured using a Shimadzu UV-2600 UV–vis spectrophotometer equipped with an integrating sphere. The integrating sphere enables an accurate determination of total reflectance and transmittance by collecting both scattered and specular reflected light at all angles. To prevent interference between reflected and transmitted signals, they were measured in separate geometrical configurations, so that the sample was placed at different positions within the integrating sphere to isolate each contribution. In the reflectance configuration, only light reflected from the sample was collected, while transmitted light was excluded; conversely, in the transmittance configuration, only light passing through the sample entered the sphere. Absorbance (*A*) was calculated using the relation *A* = 1 – (*R* + *T*), where *R* and *T* represent the total reflectance and transmittance, respectively. A BaSO_4_ plate and a glass slide were used as reference standards throughout the measurements.

### Time-Resolved Measurements

The time-resolved optical measurements were performed with the output of the collinear optical parametric amplifier (OPA, Light Conversion Orpheus HP), seeded by the amplified Yb:KGW laser system (Light Conversion Pharos). The OPA delivers 150 fs pulses in a 600 kHz train, widely tunable from UV to the infrared. For the experiments on the electron dynamics in AuNSs, the output of the OPA at a wavelength of 800 nm was used as a pump, corresponding to the maximum of their plasmonic response, while the frequency-doubled output of the ytterbium amplifier at 514 nm (100–150 fs pulse duration) was used as a probe light, as it is highly sensitive to the direct interband transitions in gold.^25^ In some measured pump–probe traces, in addition to the transient absorption signal, a short symmetric peak was visible at time-zero, interpreted as a coherent artifact due to the degenerated four-wave-mixing process. The temporal width of this feature coincides with the width of pump–probe cross-correlation, and it was fitted by the corresponding Gaussian envelope. For both bare PHI and AuNSs-PHI systems, an additional artifact was visible at 14 ps delay, which represented a repeat of the transient trace due to back reflection from the glass slide: the observed delay matches the one corresponding to the double trip of the probe beam through the substrate. This artifact was explicitly incorporated into the fitting algorithm. The direct photoexcitation of the PHI was realized with the output of the OPA tuned to a wavelength of 400 nm, above the direct interband transition offset of the PHI, with the same probe light used to monitor changes on picosecond to nanosecond time scales.
